# Membranous nephropathy without vacuolated podocytes in Fabry disease treated with agalsidase-β and carbamazepine: A case report

**DOI:** 10.1097/MD.0000000000028830

**Published:** 2022-02-18

**Authors:** Takahiro Kanai, Takane Ito, Jun Aoyagi, Takanori Yamagata

**Affiliations:** Department of Pediatrics, Jichi Medical University, 3311-1 Yakushiji, Shimotsuke, Tochigi, Japan.

**Keywords:** carbamazepine, enzyme replacement therapy, Fabry disease, membranous nephropathy, proteinuria

## Abstract

**Rationale:**

Vacuolated podocytes are the most common form of renal damage in Fabry disease, but other types of renal damage have been reported, such as membranous nephropathy (MN) or IgM nephropathy. Enzyme replacement therapy (ERT) is effective at preventing renal damage, but the nephropathies require appropriate treatment to prevent renal damage.

**Patient concerns:**

A 22-year-old male with Fabry disease presented with proteinuria during ERT with agalsidase-β and carbamazepine. He had received the treatment for 10 years and maintained normal plasma globotryaosylceramide levels.

**Diagnosis:**

Renal biopsy revealed MN without vacuolated podocytes. Immunofluorescent staining of the IgG subclass revealed granular patterns of IgG1, G2, G4, and C3 deposition in the glomerular basement membrane.

**Interventions:**

The carbamazepine dose was reduced from 600 mg/day to 200 mg/day (serum concentration 10.0-11.0–4.0–5.0 μg/mL).

**Outcomes:**

After reducing the carbamazepine dose, proteinuria was negative, and the patient has had a normal urinalysis for 17 months. Plasma globotryaosylceramide levels have also remained normal.

**Lessons:**

This report is a reminder of the co-existence of MN without vacuolated podocytes in Fabry disease during ERT with agalsidase-β and carbamazepine.

Physicians should be aware of this form of renal damage in Fabry disease, even during treatment.

## 1 Introduction

Fabry disease (MIM ID #301500) is an X-linked glycosphingolipidosis caused by deficient synthesis of the enzyme α-galactosidase A, which causes accumulation of globotryaosylceramide (GL-3) on each cell in the human body.^[1]^ Accumulation of GL-3 in podocytes in the kidney leads to vacuolated podocytes, resulting in renal damage and proteinuria. Therefore, vacuolated podocytes are the most common form of renal damage in Fabry disease. Enzyme replacement therapy (ERT) is effective at preventing renal damage. However, other types of renal damage with vacuolated podocytes have been reported in Fabry disease, such as membranous nephropathy^[2]^ (MN) and IgM nephropathy.^[3]^ These types of nephropathies require appropriate treatment to prevent renal damage.

Here, we present the first case of Fabry disease with MN without vacuolated podocytes during ERT with agalsidase-β and carbamazepine. After reducing the carbamazepine dose, the proteinuria resolved.

This study was approved by the Ethics Committee of Jichi Medical University with the patient consent statement. Written informed consent was obtained from the individual participant in this report.

## 2 Case report

**Figure 1:**
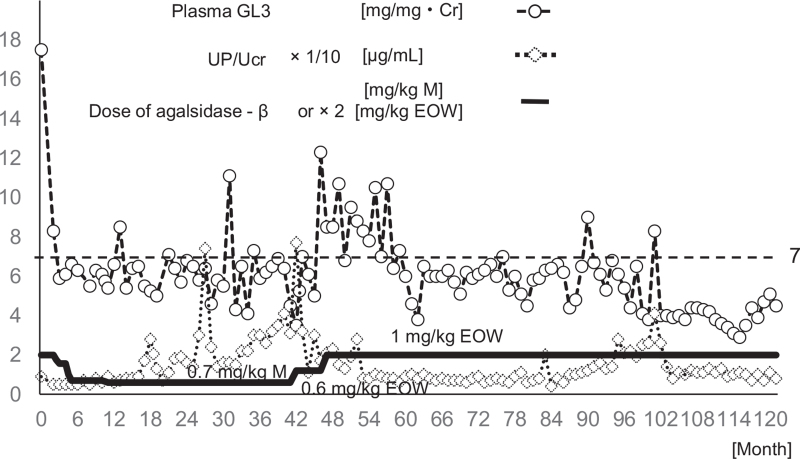
Changes in plasma GL-3 and UP/UCr levels and agalsidase-β dose over 10 years. GL-3 = globotriaosylceramide, UP = urinary protein, UCr = urinary creatinine, M = month, EOW = every other week.

**Figure 2:**
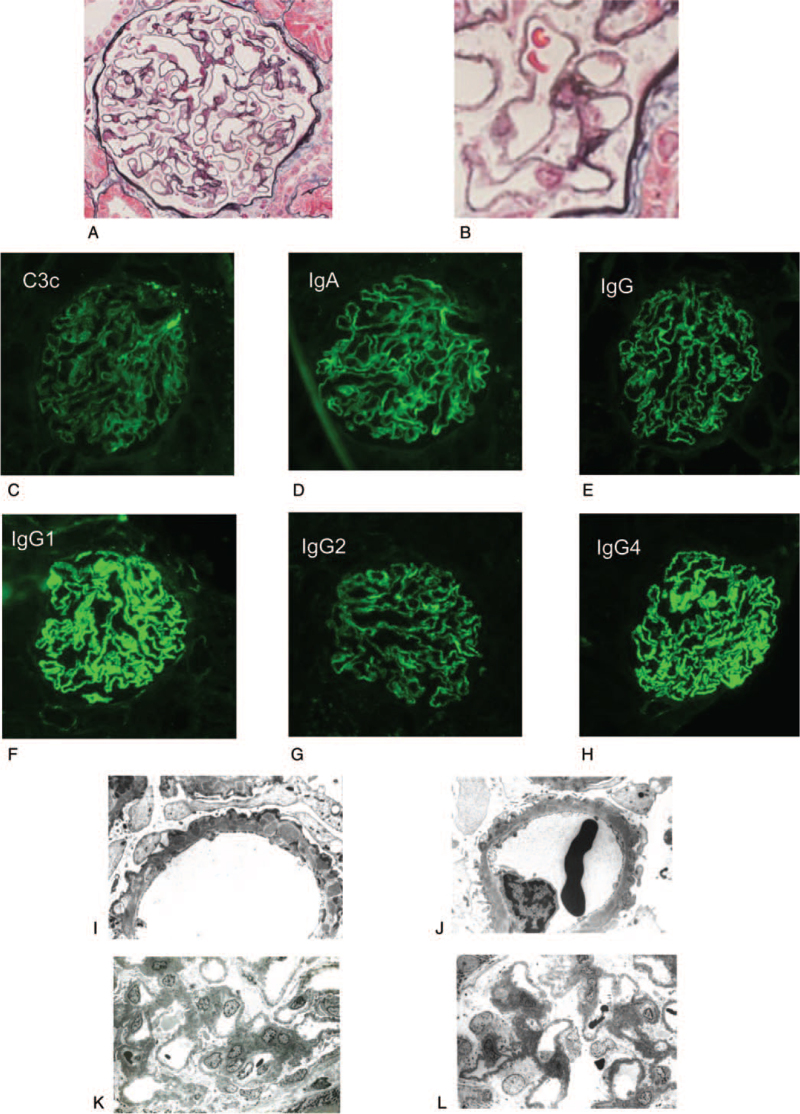
Images of renal pathology from that patient at 101st month. (A, B) Light microscopy showed a thickened glomerular basement membrane, deposits, spikes, and chain-like appearance with periodic-methenamine silver (PAM) stain. Vacuolated podocytes were not observed at 400× (a) and original magnification, 600× (b). (C–H) Immunofluorescence showed granular staining of IgG (IgG1, IgG2, and IgG4), IgA, and C3 in the glomerular basement membrane. (I–L) Electron microscopy showed intramembranous deposits and spikes in the capillary wall. No GL-3 accumulation or whorled inclusions were observed in podocytes. 6000× magnification (I,J) and 1200 × magnification (K,L).

A 22-year-old male with classic Fabry disease had been treated with both agalsidase-β and carbamazepine since he was 13 years old (Fig. 1).^[4]^ When he was diagnosed with Fabry disease at 13 years old, his renal biopsy demonstrated diffuse global vacuolated podocytes.^[4]^ He has a point mutation (Cys382Tyr) on exon 7 in the α-galactosidase A gene, which is associated with low α-galactosidase A activity (0.1 nmol/mg protein/h; reference level, 50–110 nmol/mg protein/h). He has received 1 mg/kg agalsidase-β every other week and kept normal plasma GL-3 levels since the 60th month except twice: 9.0 μg/mL at month 90 and 8.3 μg/mL at month 101 (Fig. 1). He also received carbamazepine (600 mg/day, serum concentration 10–13 μg/mL) to control his acroparesthesia. He has had normal electrocardiograms, no hyper cardiomyopathy, and normal magnetic resonance images of the brain since he was 13 years old. At month 66, his renal biopsy showed no vacuolated podocytes or accumulation of GL-3 in his kidney, with normal urinalysis.^[5]^ However, since month 95, he had presented with mild proteinuria (urinary protein/urinary creatinine [UP/UCr] of 0.21 to 0.41 g/gCr) without hematuria (Fig. 1). To examine the cause of the proteinuria, renal biopsy was performed at month 101. At that time, his height was 167.3 cm, his body weight 67.3 kg, and his blood pressure 136/87 mm Hg. He showed no edema but had slight acroparesthesia when he took a bath. His blood test showed a normal GL-3 level of 3.8 but mild proteinuria (UP/UCr 0.26 g/gCr) without hematuria (Table 1). His renal biopsy demonstrated diffuse glomerular basement membrane (GBM) thickening with spikes under the light microscope on observed 21 glomeruli, and granular GBM staining of IgG1, G2, G4, and C3, as well as IgA, was observed by immunofluorescence (Fig. 2A–H). IgG3, IgM, and C1q immunofluorescence was negative. Electron microscopy showed electron-dense deposits in the GBM (Fig. 2I–L). These findings are compatible with stage III MN. No vacuolated podocytes and no whorled inclusions were observed in podocytes by light microscopy or electron microscopy respectively.

**Table 1:** Blood and urine sample test result at renal biopsy, 102nd month.

Blood test					
WBC	4300	/μL	TP	7.0	g/dL
RBC	512 × 10^4^	/μL	Alb	3.8	g/dL
Hb	15.0	g/dL	BUN	10	mg/dL
Plt	33.2 × 10^4^	/μL	Cr	0.56	mg/dL
			UA	5.0	mg/dL
CarbamazepineCBZ	11.6	μg/mL	T.bil	0.40	mg/dL
			AST	19	U/L
GL-3	4.0	μg/mL	ALT	21	U/L
Anti-agalsidase-β IgG titer	800	titer	LDH	161	U/L
			Na	141	mmol/L
			K	4.0	mmol/L
			Cl	104	mmol/L
			Ca	9.4	mg/dL
			P	3.4	mg/dL

Urinalysis		
UP	1+	
Gluc	–	
OB	–	
Sediment		
RBC	1<	/HPF
UP	43	mg/dL
UP/UCr	0.26	mg/mg·Cr

AST = aspartate aminotransferase, ALT = alanine aminotransferase, Alb = albumin, BUN = blood urea nitrogen, Cr = creatinine, Gluc = glucose, Hb = hemoglobin, LDH = lactate dehydrogenase, OB = occult blood, Plt = platelet, RBC = red blood cell, TP = total protein, T.bil = total bilirubin, UA = uric acid, UP = urinary protein, UCr = urinary creatinine, WBC = white blood cell.

Judging from the staining of IgG subtypes, drugs were suspected as the cause of MN. Agalsidase-β is an essential drug for Fabry disease. Therefore, the carbamazepine dose was reduced from 600 mg/day to 200 mg/day (serum concentration 10.0–11.0 to 4.0–5.0 μg/mL). After reducing the carbamazepine dose, the proteinuria resolved (UP/UCr,0.14 g/gCr) by the 103rd month and the patient has maintained normal urinalysis and normal renal function for 17 months. However, the reduction of the carbamazepine dose led relapse of acroparesthesia and pregabalin (300 mg/day) was added at month 103 to relieve it.

## 3 Discussion

To the best of our knowledge, this is the first report of MN without vacuolated podocytes in Fabry disease, and the proteinuria was resolved by reducing the carbamazepine dose. Vacuolated podocytes are one of the most common forms of renal damage in Fabry disease,^[6]^ but physicians should be aware that MN is also a form of renal damage in Fabry disease during ERT with agalsidase-β and carbamazepine.

MN is caused by GBM deposits of immune complexes comprised of antigen-antibody complexes. The antigen-antibody complexes may be of unknown etiology or related to another disease or drugs, causing idiopathic MN or secondary MN, respectively. Idiopathic MN usually presents predominantly as positive IgG4 staining, whereas secondary MN presents as positive IgG1and IgG2 staining, as well as positive IgG4 staining,^[^7^,^^8^^]^ which is compatible with our case. In secondary MN, removing the causal factors in the deteriorated condition is an effective treatment. In Fabry disease, ERT is an essential and critical treatment to metabolize GL-3. Therefore, in our patient, the carbamazepine dose was reduced. We cannot conclude whether only carbamazepine itself or the combination of agalsidase-β and carbamazepine caused the MN. Furthermore, the IgG deposited in the GBM could not be confirmed as being related to agalsidase-β or carbamazepine, but there are 2 prior reports of MN being induced during carbamazepine treatment. In both cases, the proteinuria was negative after reducing the carbamazepine dose.^[^9^,^^10^^]^ On the other hand, there has been no report of agalsidase-β causing MN and it is difficult to reduce the amount of enzyme replacement because several reports have shown that this can cause renal damage.^[^5^,^^11^^]^

Therefore, it is important for physicians to know that proteinuria was resolved after reducing the carbamazepine dose in MN during ERT with agalsidase-β and carbamazepine for Fabry disease.

In conclusion, this report demonstrates that MN is a form of renal damage in Fabry disease during ERT with agalsidase-β and carbamazepine. The proteinuria was resolved after reducing the carbamazepine dose.

## Author contributions

**Conceptualization:** Takahiro Kanai.

**Formal analysis:** Takahiro Kanai.

**Funding acquisition:** Takahiro Kanai.

**Investigation:** Takahiro Kanai.

**Project administration:** Takahiro Kanai, Takane Ito, Jun Aoyagi.

**Supervision:** Takanori Yanmagata.

**Writing – original draft:** Takahiro Kanai.

**Writing – review & editing:** Takahiro Kanai.
